# Melatonin decreases in vitro viability and migration of spheres derived from CF41.Mg canine mammary carcinoma cells

**DOI:** 10.1186/s12917-019-2142-z

**Published:** 2019-11-04

**Authors:** Consuelo Serrano, Sofía Guzmán, Jose Ignacio Arias, Cristian Gabriel Torres

**Affiliations:** Laboratory of Biomedicine and Regenerative Medicine, Department of Clinical Sciences, Faculty of Veterinary and Animal Sciences, Universidad de Chile, Santa Rosa 11735, 8820808 La Pintana, Chile

**Keywords:** Canine cancer cells, Mammary cancer stem cells, Melatonin

## Abstract

**Background:**

Mammary cancer is a common disease affecting female dogs, where approximately 50% of the cases are malignant. There is a subpopulation of cancer cells with stem cell-like features within the tumour microenvironment, which can form in vitro spheres, cell structures that grow in anchor-free conditions. This cell population shows resistance to conventional antitumor treatments explaining in part the recurrence of some type of cancers. It has been previously reported that spheres derived from CF41.Mg canine mammary carcinoma cells exhibit several stemness features. Melatonin has shown antitumor effects on cancer mammary cells; nevertheless, its effects have been poorly evaluated on canine mammary cancer stem-like cells. In this regard, it has described that melatonin decreases the expression of OCT-4 in CMT-U2229 mammary cancer cells, a transcription factor that participates in the modulation of self-renewal and drug resistance in cancer stem-like cells. The aim of this study was to compare the effects of melatonin on viability and migration of canine mammary carcinoma CF41.Mg-spheres, and CF41.Mg-parental cells. CF41.Mg cells were grown in DMEM high-glucose medium containing 10% bovine foetal serum. CF41.Mg-spheres were cultured in ultra-low attachment plates with serum-free DMEM/F12 containing several growth factors. Cell viability (MTS reduction) and migration (transwell) assays were conducted in presence of melatonin (0.01, 0.1 or 1 mM).

**Results:**

Melatonin decreased cell viability at 1 mM (*P* < 0.05), with a significant reduction in spheres compared to parental cells at 24 and 48 h (*P* < 0.05). Cell migration was inhibited in response to non-cytotoxic concentration of melatonin (0.1 mM) (*P* < 0.05) in spheres and monolayer of cells, no significant differences were detected between both cell subtypes.

**Conclusions:**

These results indicate that melatonin reduces viability and migration of CF41.Mg cells, where spheres exhibit greater sensitivity to the hormone. Thus, melatonin represents a valuable potential agent against mammary cancer cells, especially cancer stem-like cells.

## Background

Mammary tumours represent the neoplasms most frequently diagnosed in reproductively intact female dogs [1, 2]. Surgery is the treatment of choice for this disease; however, this therapeutic option is not feasible in the case of non-resectable or metastatic tumours [3]. Adjuvant chemotherapy can be used as therapeutic alternative in cases of tumours with high probability of metastasis, however, some cancer cells may acquire resistance to commonly available drugs [4], therefore it is relevant to seek novel therapeutic alternatives.

The existence of subpopulations of cancer cells with stemness features (cancer stem-like cells (CSC)), also called tumour-initiating cells, may partially explain some characteristics of tumour progression, such as neoplastic recurrence, metastasis and drug resistance [5]. Mammary CSC exhibit the capacity for self-renewal, chemo and radioresistance, vascular mimicry, invasiveness and CD44^+^/CD24^−/low^ phenotype [4, 6]. It is possible to obtain them in vitro from cell lines by means of the spheres-forming ability, which is one of the most used strategies to isolate and identify CSC [6].

Spheres derived from canine mammary carcinoma cells CF41.Mg express in a high proportion the CD44^+^/CD24^−/low^ phenotype, in addition to other stemness characteristics such as self-renewal and relative chemoresistance to doxorubicin, paclitaxel and simvastatin [4]. On the other hand, CSC also express transcription factors associated with stemness, including OCT4, which plays a key role in carcinogenesis and provides a mechanism by which CSC could acquire or maintain a phenotype resistant to various therapies [7].

Melatonin (N-acetyl-5-methoxytryptamine) is an indole hormone synthesized by the mammalian pineal gland and other tissues such as retina, gastrointestinal tract, skin, among others [8, 9]. Physiological levels of melatonin in healthy dogs is dependent of diurnal cycle and season, rising during the night and in autumn-winter seasons. These plasma levels fluctuate between 2 and 13 pg/ml, depending on the factors already mentioned [10, 11]. On tumour cells, this liposoluble hormone has pleiotropic effects including antioxidant, anti-angiogenic, pro-apoptotic and antiproliferative effects through receptor-dependent and receptor-independent mechanisms [12, 13], nevertheless, its actions on CSC has been poorly studied [14]. Melatonin-receptors MT1 and MT2 are ubiquitous [15] G-protein-coupled receptors that once activated by their ligand induce an inhibition of adenylyl cyclase and cyclic AMP, which translates into antiproliferative effects [16–19]. Anti-tumour effects mediated by MT1-interaction correlate with antiestrogenic effects induced in oestradiol receptor type α (ERα)-positive mammary cancer cells, where melatonin represses the transcriptional activity of ERα (decreasing the phosphorylation of receptor and/or coactivator molecules) and inhibits aromatase activity [16, 17]. Thus, this hormone acts as an inhibitor of cell proliferation and an inducer of apoptosis, decreasing the mitogenic response of tumour cells to oestradiol [20]. In this regard, it has been described that melatonin reduces ERα and OCT4 expression and the binding of the ERα to OCT4, down-regulating sphere-forming ability in oestradiol-dependent cells, which suggest that this hormone could modulate self-renewal in CSC [21]. There is evidence that melatonin decreases OCT4 immunoexpression in spheroids derived from canine mammary cancer cells CMT-U2229 [7], which may partially explain the antiproliferative effect induced by this hormone on these cells [7, 13]. Melatonin also decreases invasiveness ability of CMT-U2229-spheres and modulates epithelial-mesenchymal transition (EMT) [7]. Nevertheless, it is necessary to study its in vitro antitumor effects in other cell types that are more representative of mammary tumours and with higher malignancy. This is the case of CF41.Mg cell line, which exhibit an invasive and metastatic phenotype [13, 22]. The aim of this work was to determine the in vitro effect of melatonin on the viability and migration of CSC derived from CF41.Mg canine mammary carcinoma cells.

## Results

**Fig. 1:**
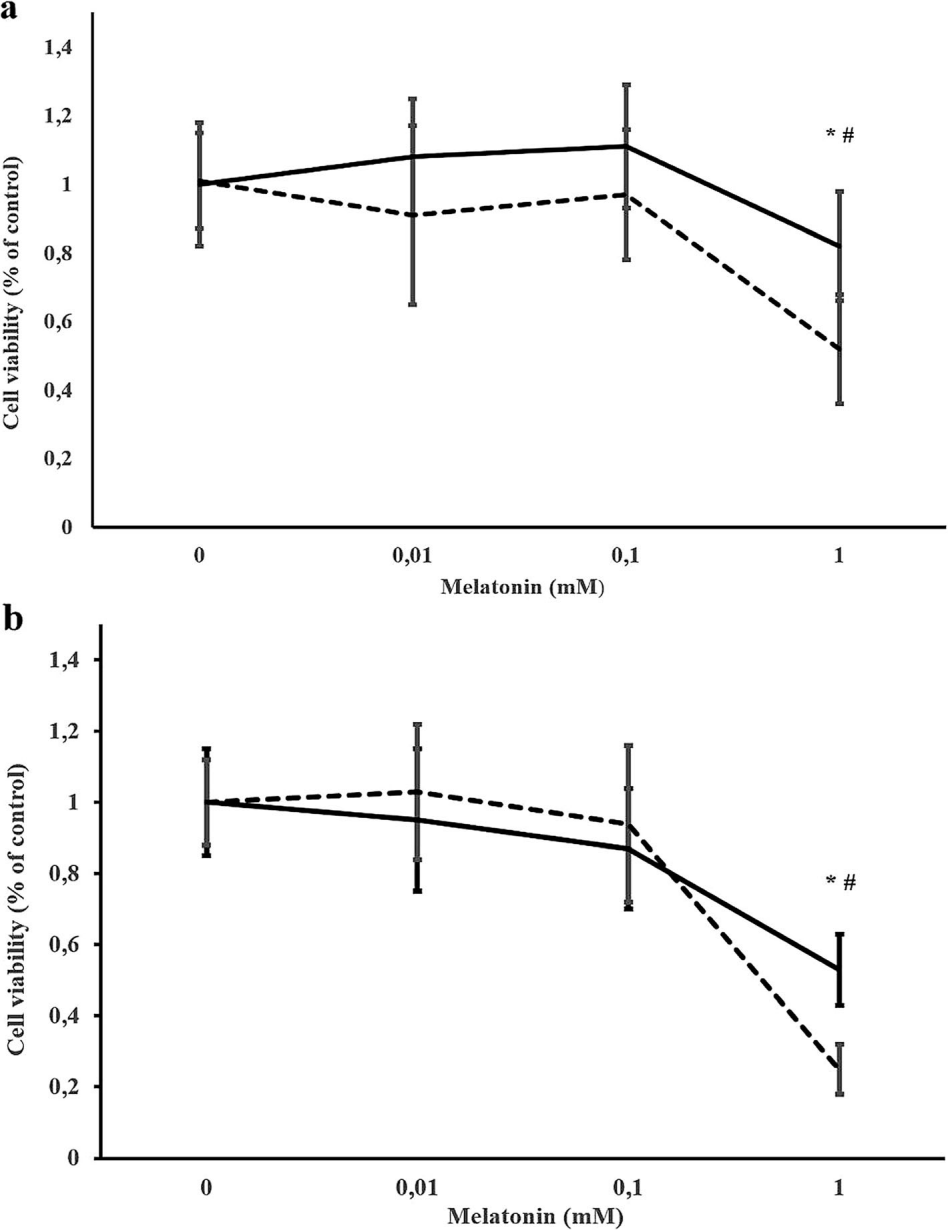
Melatonin decreases viability of spheres derived from CF41.Mg canine mammary carcinoma cells. CF41.Mg cells in monolayer (solid line) and CF41.Mg-spheres (dashed line) were treated with different concentrations (0–1 mM) of melatonin for 24 (**a**) and 48 (**b**) h. The proportion of viable cells was determined by MTS assay. Values are mean ± SD of 3 individual experiments in triplicate. * *P* < 0.05 when comparing different experimental groups; ^#^ *P* < 0.05 when comparing both types of cultures

**Fig. 2:**
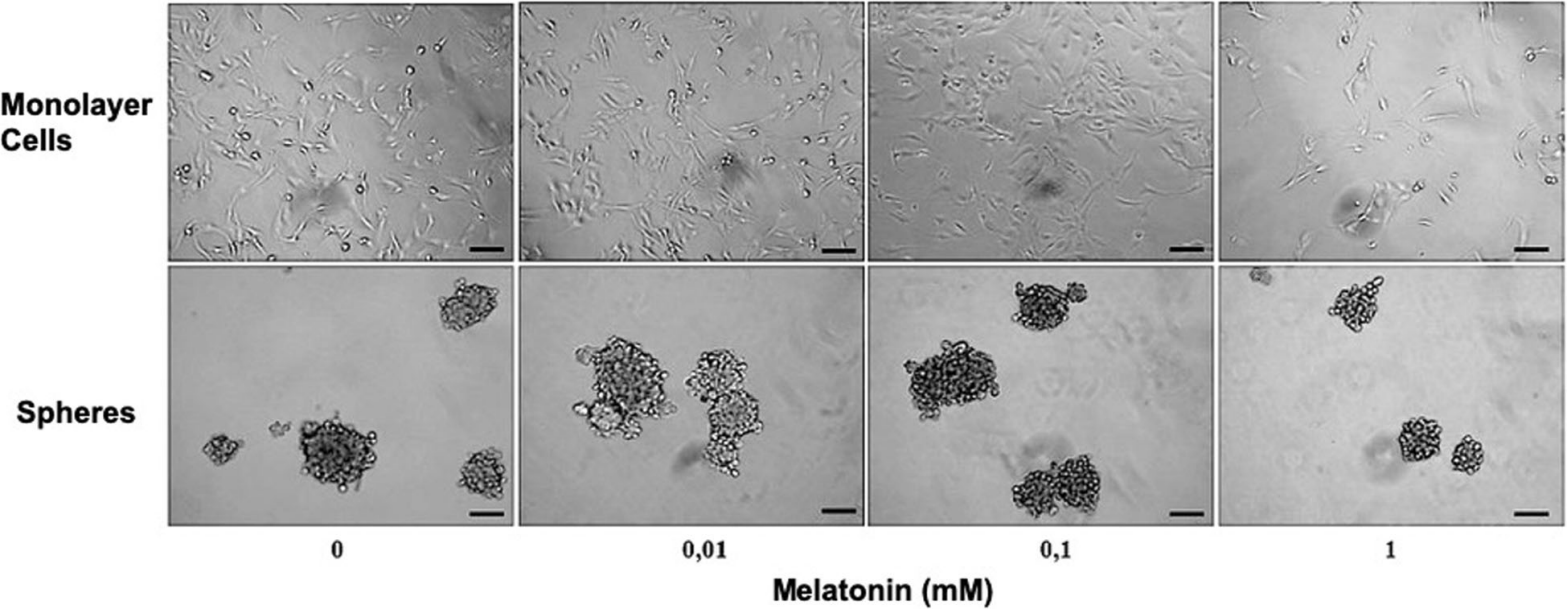
Representative photomicrographs of effect of melatonin (0-1 mM) on CF41.Mg monolayer cells and spheres viability at 48 h. Scale bar: 100 μm

**Fig. 3:**
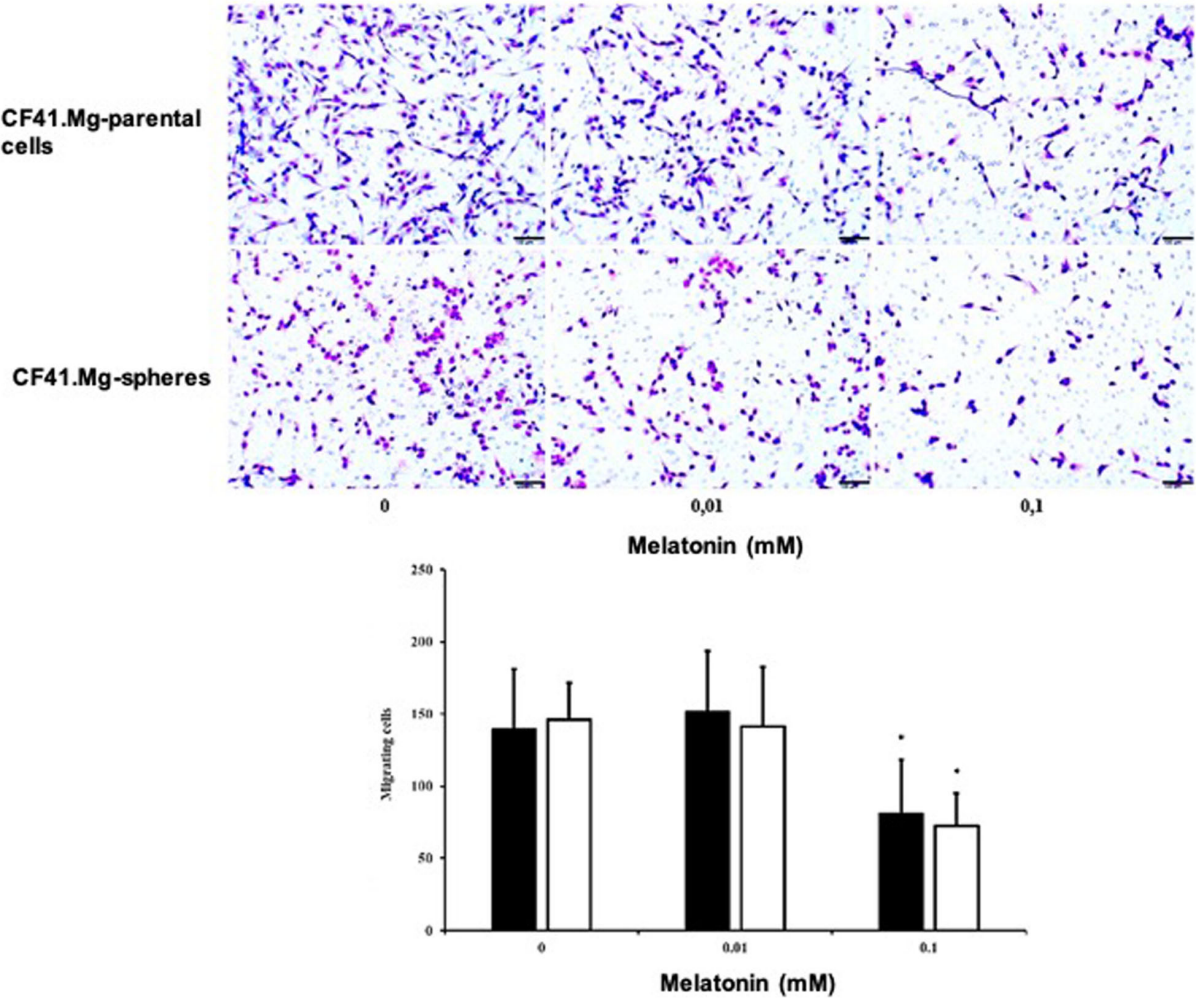
Melatonin decreases migration ability of spheres derived from CF41.Mg canine mammary carcinoma cells. Representative photomicrographs of migration ability in CF41.Mg monolayer of cells and CF41.Mg-spheres in response to non-cytotoxic concentrations of melatonin (0–0.1 mM) for 24 h. The migrating cells were stained with Giemsa and counted in a light microscope. Scale bars: 100 μm. Histogram quantifies migrating CF41.Mg cells (black) and CF41-Mg-spheres (white). Means ± SD of three independent transwells in duplicate. *P < 0,05 compared with the control cells

Viability of CF41.Mg cells was reduced (*P* < 0.05) in both monolayer (53.4 ± 9.9% of viability compared to control at 48 h) and spheres (52.1 ± 15.8% and 25.4 ± 6.7% compared to control at 24 and 48 h respectively) supplemented with 1 mM melatonin. No significant differences were detected at lower concentrations (0.01 and 0.1 mM). When comparing the viability of both cultures in response to 1 mM melatonin, spheres viability decreased more intensely (*P* < 0.05) than monolayer of cells at both time points (Figs. 1 and 2). On the other hand, the number of migrating cells was reduced (*P* < 0.05) after 24 h of incubation in medium supplemented with of 0.1 mM of melatonin (CF41.Mg = 81 ± 37; and CF41.Mg-spheres = 72 ± 23) compared to 0.01 mM melatonin (CF41.Mg = 152 ± 42; and CF41.Mg-spheres = 141 ± 41) and vehicle (CF41.Mg = 140 ± 42; and CF41.Mg-spheres = 146 ± 25) (Fig. 3). No significant differences were detected in the migration ability between monolayer and spheres.

## Discussion

A key feature of CSCs is its potential to resist conventional anti-tumour chemo and radiotherapy, a condition already observed in CSCs derived from canine mammary cancer [5, 6]. It is well known that chemo-resistance is acquired by a variety of mechanisms including high expression and function of multidrug-resistance (MDR) transport proteins involved in the excretion of xenobiotics [4]. In accordance with previous studies [9, 12, 13], our data indicates that melatonin supplementation in culture medium decreases viability and migration of CF41.Mg cells at concentrations of 1 and 0.1 mM respectively, being its greatest effect on CSC viability. There is currently little evidence on the effect of melatonin on CSC biology and the mechanism of action involved; however, it has been suggested that this hormone may target the transcription factor OCT4 [7], modifying its function on self-renewal, pluripotency and drug resistance [21].

There is evidence that CSCs is a heterogeneous population where two morphological patterns are recognized, one mesenchymal (exhibiting a CD44^+^/CD24^−/low^ phenotype) and another epithelial (ALDH^+^ phenotype). The first, usually is in a quiescent state and can be induced by oxidative stress, which can be pharmacologically modulated. In contrast, epithelial-CSCs are in a more proliferative state [23, 24]. Data of our lab suggest that CF41.Mg-spheres exhibit a mesenchymal phenotype (CD44^+^/CD24^−/low^ phenotype, low proliferative rate) [4], condition that may be inhibited by melatonin, since its antioxidant effect [12], explaining in part the cell viability data shown here. As just described, it has reported that melatonin decreases E-cadherin and increases vimentin and N-cadherin expression in both canine and human mammary tumour cells (CMT-U229 and MCF7 respectively), inducing an anti-invasive effect in these cells [7]. It is relevant to consider that CMT-U229 is a cell line derived from a benign mixed mammary tumour [13], unlike CF41.Mg cell line used in this study, which is representative of high histological and metastatic canine mammary tumours [13, 22]. Thus, overall our data and previously reported information suggest that melatonin exerts an antiproliferative effect on canine mammary CSCs.

In general, exogenous administration of melatonin is safe in dogs because it almost does not induce side effects, although the hormone can cause sedation in a low proportion of animals [25]. In humans, oral melatonin improves non-restorative sleep and circadian rhythm amplitudes [26], therefore the pharmacological use of this hormone seems innocuous.

Melatonin may represent a promising adjuvant option against high malignancy canine mammary carcinomas, nevertheless, it is necessary to outspread these analyses to other mammary tumour cells. Additional in vitro studies with lower concentrations of melatonin that mimic the plasma concentration achieved in dogs receiving a routine therapeutic dose (total posology of 3–6 mg twice a day [25]) are required. It has described that melatonin plasma bioavailability is dose dependent [27], where the maximum plasma concentration reached after an oral dose of 2 mg is 1.15 ± 0.92 ng/ml [28].

Thereby, this study reinforces the in vitro therapeutic potential of melatonin, especially on cells that have a chemo-resistant phenotype.

## Conclusion

Melatonin reduces viability and migration of CF41.Mg cells, where CF41.Mg-CSC exhibit a greater proliferative sensitivity to this hormone. Thus, melatonin may be considered a potential anti-tumour agent against canine mammary stem-like cells, which supports future clinical trials.

## Methods

### Cell culture

CF41.Mg canine mammary carcinoma cells (CRL-6232; ATCC®, Manassas, VA, USA) were cultured in adherence conditions with high glucose-DMEM (4.5 g/L D-Glucose; Hyclone, GE Healthcare Life Sciences, Logan, UT, USA) supplemented with 10% foetal bovine serum (FBS) (Hyclone, GE Healthcare Life Sciences, Logan, UT, USA), 100 μg/mL streptomycin, 100 IU/mL penicillin and 0.25 μg/mL amphotericin B. Spheres derived from CF41.Mg cells were grown in ultralow attachment plates (Corning, NY, USA) containing culture medium serum-free DMEM/F12 (Sigma-Aldrich, Saint Louis, MO, USA) plus 10 ng/mL basic fibroblastic growth factor (bFGF) (Life Technologies Corp, Carlsbad, CA, USA) 10 ng/mL epidermal growth factor (EGF) (Life Technologies Corp, Carlsbad, CA, USA), 5 μg/mL insulin (Sigma-Aldrich, Saint Louis, MO, USA), 4 μg/mL heparin (Sigma-Aldrich, Saint Louis, MO, USA), 2% B27 (Life Technologies Corp, Carlsbad, CA, USA) and, 20 μg/ml penicillin, 20 μg/mL streptomycin and 0.05 μg/mL amphotericin B (Corning, NY, USA). All cultures were maintained in a humidified atmosphere with 5% CO_2_ at 37 °C.

### Viability assay

CF41.Mg cells (2 × 10^3^ cells/well into 96-well plates) and CF41.Mg-spheres (5 × 10^3^ cells/well into non-adherent 96-well plates) were seeded in triplicate. After 24 h, cells were incubated for 24 and 48 h in culture medium supplemented increasing concentrations of melatonin (0.01, 0.1, 1 mM) (Selleckchem, Houston, TX, USA). In order to determine the viability, cells were exposed to 3-(4,5-dimethylthiazol-2-yl)-5-(3-carboxymethoxyphenyl)-2-(4-sulfophenil)-2H-tetrazolium (MTS) for 3 h at 37 °C and the retained dye was measured with a microplate reader (Biotek Instruments, Winoosky, VT, USA) at 490 nm. Cell viability, referred to as the proportion of live cells at the end of the experiment, was calculated as a relative value in relation to the non-stimulated control, where the control group was considered to be 100% viability. Three independent experiments were performed.

### Migration assay

Migration assays were carried out using Costar migration chambers (Transwell® 8-μm pore size, 24-wells; Costar, Kennebunk, ME, USA). CF41.Mg monolayer cells and spheres (5 × 10^4^) were incubated in presence of 0, 0.01, 0.1 mM melatonin for 24 h in duplicate against a gradient of 5% FBS. Non-migrating cells were wiped from the upper side of the transwell membrane, and the migrating cells were fixed with cold methanol, and stained with Giemsa 1%. Six fields were randomly selected and counted in each transwell under a light microscope at 10x magnification. Three independent experiments were carried out.

### Statistical analysis

The Shapiro-Wilk test was used to determine data normality. ANOVA and Bonferroni or Kruskal Wallis tests were carried out to determinate differences between experimental groups. Mann-Whitney U test was used to define differences between cell subtypes. A value of *P* < 0.05 was considered statistically different. Data was analysed using Infostat statistical software (Córdoba, Argentina).

## Data Availability

The datasets used and/or analyzed during the current study are available from the corresponding author on reasonable request.

## Abbreviations

ALDH: Aldehyde dehydrogenase
AMP: Adenosine monophosphate
ANOVA: Variance analysis
B27: Neuronal cell culture serum-free supplement
bFGF: Basic fibroblastic growth factor
CD24: Heat stable antigen CD24
CD44: Homing cell adhesion molecule
CSC: Cancer stem-like cells
DMEM: Dulbecco’s modified eagle medium
EGF: Epidermal growth factor
ERα: Oestradiol receptor α
FBS: Foetal bovine serum
MDR: Multidrug resistance
MT: Melatonin receptor
MTS: (3-(4,5-dimethylthiazol-2-yl)-5-(3-carboxymethoxyphenyl)-2-(4-sulfophenyl)-2H-tetrazolium)
OCT4: Octamer-binding transcription factor 4
